# “A single viral video can undo months of health education”: social media, trust, and vaccine hesitancy in wartime Ukraine

**DOI:** 10.1186/s12939-026-02845-8

**Published:** 2026-04-18

**Authors:** Safiyeh Tayebi, Ana Lorena Ruano, Liudmyla Ishchenko, Fedir Lapii, Iuliia Pavlova, Oleski Korzh, Olga Belousova, Ubydul Haque

**Affiliations:** 1 Department of Geography, Rutgers, The State University of New Jersey, 54 Joyce Kilmer Avenue, Piscataway, New Jersey 08854-8045 USA; 2 Center for International Health, Department of Global Health and Primary Care, University of Bergen, Bergen, Norway; 3 Ukrainian Laboratory of Quality and Safety of Agricultural Products (Ishchenko), Kyiv, Ukraine; 4 Department of Pediatrics, Immunology, Infectious and Rare Diseases, European Medical School, International European University, Kyiv, Ukraine; 5 Department of Theory and Methods of Physical Culture, Ivan Boberskyj Lviv State University of Physical Culture, Lviv, 79007 Ukraine; 6 Department of General Practice—Family Medicine, Kharkiv National Medical University, Kharkiv, Ukraine; 7 Faculty of Medicine, Vilnius University, M. K. Čiurlionio g. 21, Vilnius, LT-03101 Lithuania; 8 Rutgers Global Health Institute, New Brunswick, NJ USA

**Keywords:** Vaccine hesitancy, Ukraine, Conflict, Trust, Health communication, Maternal decision-making

## Abstract

This study examines vaccine hesitancy in wartime Ukraine, where institutional mistrust, displacement, and emotional trauma reshape parental decision-making. Drawing on in-depth interviews with mothers and pediatric professionals, we explore how caregivers navigate immunization choices amid disrupted health systems, contested information environments, and weakened trust in state and international actors. We frame hesitancy not as a static or irrational stance but as a dynamic process shaped by context, history, and emotion. Findings reveal that hesitancy stems from intersecting structural and interpersonal factors, including the erosion of institutional credibility, fear-based digital content, and generational memories of coercive medicine. Mothers often rely on social networks and peer narratives to fill gaps left by healthcare services, while providers struggle to communicate effectively under wartime pressures. Vaccine decision-making in this context is deeply relational, moral, and affective, extending beyond biomedical considerations. Addressing hesitancy in conflict-affected settings requires public health strategies that move beyond access and education, prioritizing empathetic communication, rebuilding trust, and acknowledging the emotional and symbolic dimensions of vaccination during crisis.

## Introduction

In conflict-affected settings, routine health services like vaccines often become inaccessible [9, 38]. Populations are exposed to multiple forms of vulnerability, including trauma, insecurity, and loss of institutional tools [35]. These disruptions fragment communication and service delivery, leading to misinformation, inconsistent narratives, and systemic silence from health authorities [46]. This particularly affects vaccines, as informal networks and social media amplify and reinforce misinformation [22, 55], leading to delays in acceptance or refusal of vaccines altogether.

The war in Ukraine shows how accessing routine care can be risky, confusing, or altogether impossible [13]. Here, eroding institutional trust and capacity couples with concerns about safety or efficacy of treatments, leading to vaccine hesitancy [7, 28, 56]. The Russian invasion in 2022, the vaccine program has faced layers of disruption expressed through large-scale displacement, infrastructural damage, and the redirection of healthcare resources may have reduced access to routine immunization services across the country [11]. In regions experiencing active conflict or occupation, these disruptions were likely more severe, while in relatively safer areas, the psychological toll of ongoing threats and limited mobility may have discouraged care-seeking behaviors. At the same time, the growing role of digital platforms in health communication likely complicated efforts to promote vaccination. In such an environment, official health messaging may have struggled to compete with more emotionally engaging or misleading content circulating online [1, 21, 47].

Communication disruptions are further complicated by social and generational factors that shape how vaccine decisions are made [21]. For many younger parents, especially those raised in periods of institutional instability, trust in formal health systems may be limited [43]. Instead, decisions around vaccination may be shaped more by peer groups, digital communities, and personal experience than by institutional guidance [37]. In this context, decisions about childhood immunization may reflect not only assessments of risk and benefit, but also deeper dynamics of trust, identity, and care.

Despite the severity and complexity of these dynamics, existing literature on vaccine hesitancy in Ukraine remains limited. Scholarship has begun to document immunization coverage rates, cold chain reliability, and institutional response under wartime conditions [17, 26, 45], and other studies have explored the emotional, social, and relational aspects of vaccine behavior from the perspective of caregivers and providers [3, 18, 25, 44]. However, much of the European vaccine hesitancy literature continues to focus on high-income, politically stable countries, using frameworks that assume access, continuity, and institutional trust, assumptions that do not hold in fragile or conflict-affected states [4, 23]. The early conceptualizations of vaccine hesitancy provided an important foundation for understanding vaccination attitudes, the COVID-19 pandemic has generated a rapidly expanding body of research examining how trust, misinformation, political polarization, and institutional credibility shape vaccine decision-making. Recent studies have highlighted how pandemic conditions intensified public debates around scientific authority and individual autonomy, often reshaping perceptions of vaccine risk and institutional legitimacy. In addition, scholars have emphasized the importance of historical and regional contexts in shaping vaccination attitudes, particularly in post-Soviet and Eastern European societies where legacies of institutional distrust and health system transformation continue to influence public perceptions of medical authority. These studies suggest that vaccine hesitancy cannot be understood solely as an individual-level phenomenon but must be examined within broader social, historical, and political contexts that shape trust in public health institutions.

Where studies have addressed low-resource settings, they tend to prioritize structural constraints over the affective and cognitive dimensions of vaccine decision-making. There is an urgent need to fill this gap by incorporating qualitative evidence that centers the lived experience of vaccine decision-makers in war-affected contexts.

This study responds to that need by investigating vaccine hesitancy in Ukraine during wartime as a complex, contextually embedded phenomenon shaped by the convergence of health system fragility, trauma, social influence, and digital information disorder. Rather than framing hesitancy as a deviation from rational public health behavior, we approach it as a form of adaptive reasoning within a context of uncertainty and fear. Our goal is to develop a deeper understanding of how caregivers and healthcare professionals interpret risk, construct trust, and act or refrain from acting on behalf of children’s health in environments where conventional health systems have faltered. In doing so, we seek to inform more context-sensitive approaches to immunization strategy, provider training, and communication design, not only in Ukraine but in other fragile settings facing overlapping health and governance crises.

## Methodology

### Conceptual framework

**Fig. 1:**
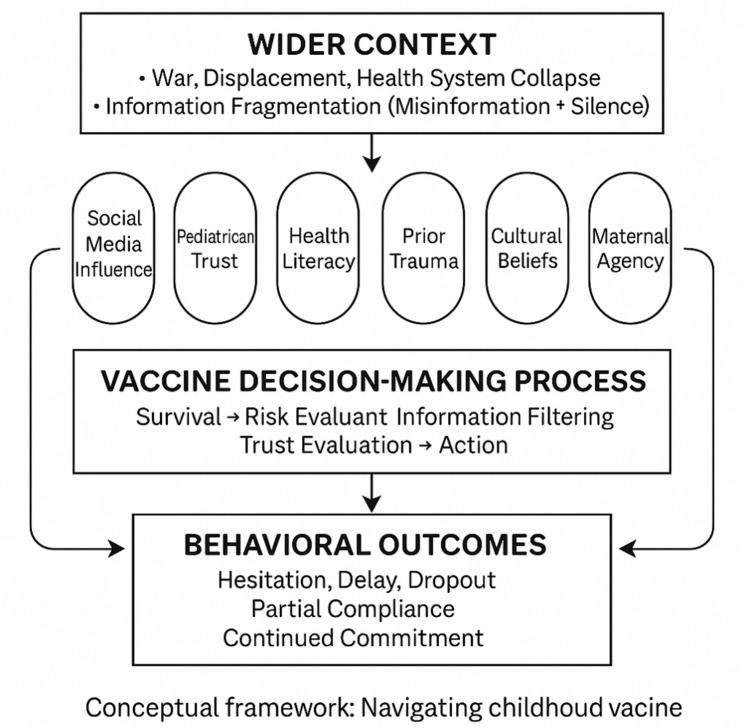
A multilevel framework of vaccine behavior

This study conceptualizes vaccine hesitancy among displaced and war-affected populations as an emergent behavior shaped by structural disruptions, socio-cultural dynamics, and individual decision-making processes [4]. The Multilevel Framework of Vaccine Behavior (see Fig. 1) is useful for understanding how broader contextual forces like war, displacement, and health system collapse interact with interpersonal influences and individual perceptions to shape vaccine-related choices and actions [4, 23]. This framework enables a layered analysis of how trust, risk perception, prior trauma, and fragmented information systems converge to influence vaccine decision-making, ultimately leading to diverse behavioral outcomes such as delay, dropout, or commitment to vaccination.

In conflict-affected settings, the wider context is characterized by war, displacement, health system collapse, the fragmentation of information through misinformation, and silence. These act as the backdrop in which vaccination decisions unfold [53, 56]. These structural conditions feed into a range of individual and social influences, including social media narratives, levels of pediatrician trust, health literacy, prior traumatic experiences, culturally embedded beliefs, and the perceived agency of mothers in making health decisions for their children [5, 12, 48]. These factors interact dynamically in the vaccine decision-making process, which involves a progression through survival concerns, risk assessment, filtering of available information, evaluation of trust, and ultimately, the decision to act [23]. Scholarship on vaccine hesitancy has also emphasized the role of parental identity, responsibility, and autonomy in shaping vaccination decisions. Research shows that some parents interpret vaccination choices as an expression of responsible and informed parenting, often framed through individualized risk assessments and moral responsibility for children’s health. In these contexts, vaccine decisions are not simply medical choices but are embedded in broader cultural expectations of parental care and vigilance. Complementing this perspective, highlight in a mandatory environment how narratives of health empowerment and self-determination shape vaccination debates, with some parents framing vaccine refusal or delay as a form of autonomy and personal agency in health governance. The outcomes of this process manifest behaviorally, ranging along a spectrum that includes hesitation, delay, dropout, partial compliance, and continued commitment to vaccination. Importantly, these behavioral outcomes may in turn reinforce or reshape the factors feeding into future decisions, creating a feedback loop that sustains or shifts patterns of vaccine behavior over time.

Building on the conceptual framework, this study examines how vaccine hesitancy emerges and is negotiated within the complex social and institutional conditions of wartime Ukraine. While much of the existing literature focuses on individual beliefs or misinformation, this study seeks to understand vaccine decision-making as a socially embedded process shaped by trust, institutional disruption, and lived experiences of conflict. The primary research question guiding this study is: How do mothers and healthcare professionals in Ukraine interpret and negotiate childhood vaccination decisions under wartime conditions?

### Data collection

We conducted 68 in-depth interviews: 46 with mothers of children and 22 with medical professionals across localities, or oblasts, in Ukraine, including both conflict-affected and relatively stable regions. Recruiting participants during an ongoing war posed practical challenges. Although a flexible and adaptive sampling strategy was used to reach diverse participants, some perspectives may not have been captured. We employed a purposive sampling strategy, combining snowball, maximum variation, and criterion-based approaches. Snowball sampling was used to identify eligible participants, particularly given the challenges of recruitment during wartime conditions. To ensure a broad range of perspectives, we simultaneously applied maximum variation sampling, capturing diversity across geographic regions (urban/rural), exposure to conflict (frontline vs. stable zones), professional roles (mothers vs. healthcare providers), and socioeconomic status. Eligibility criteria served as a form of criterion sampling: mothers were included if they had at least one child, resided in Ukraine during the war (2022–2024), and had made at least one vaccine-related decision during that period. Physicians were eligible if they provided pediatric care during the same timeframe. Sampling continued until thematic saturation was reached. Interviews were conducted in Ukrainian by a Ukrainian interviewer (LI) via video conferences. A semi-structured guide was developed based on prior studies and pilot interviews, covering themes such as vaccination behaviors, trust in healthcare, war-related disruptions, information sources, and institutional accountability.

Interviews ranged from 15 to 50 min and were audio-recorded, transcribed verbatim, and translated into English for analysis. All identifying information was removed to ensure confidentiality.

### Data analysis

This study employed a qualitative approach to explore perceptions and experiences related to childhood vaccination among mothers and healthcare professionals in Ukraine. The goal was to understand the underlying fears, motivations, and contextual influences that shape vaccine-related decisions, particularly during times of systemic stress and conflict.

Iterative thematic analysis was employed to analyze the qualitative data generated from interviews with mothers and pediatric healthcare professionals. The analysis followed a structured yet flexible approach, allowing for both theory-informed and emergent insights. The process began with multiple close readings of the translated and transcribed interviews to achieve immersion in the data. This familiarization phase allowed the research team to attune themselves to the emotional, contextual, and behavioral nuances in participant narratives, especially as they related to vaccination decisions in a post-conflict setting (ST).

In the second step, open coding was conducted manually and iteratively. Initial codes were developed inductively from the data, capturing distinct ideas, experiences, and expressions of concern or motivation. These included references to vaccine side effects, healthcare provider interactions, media influences, and disruptions caused by war. As the coding progressed, refinements were made to capture both individual variation and thematic consistency across interviews (ST, ALR).

In the third stage, related codes were grouped into broader conceptual categories to organize the data into interpretable clusters. These categories reflected shared meanings across participants and captured intermediate-level constructs such as institutional trust, perceived vaccine harm, information-seeking behavior, and structural barriers to access (ST, ALR). At this stage, analytical memos were used to track evolving interpretations and linkages between categories.

The fourth step involved identifying overarching themes that synthesized the categories into coherent narratives. This stage incorporated a hybrid analytical lens by drawing selectively on the Theoretical Domains Framework (TDF) to structure and interpret patterns where appropriate. TDF domains such as knowledge, social influences, environmental context, and beliefs about consequences, offered a useful theoretical scaffold for comparing perspectives between mothers and healthcare professionals. However, the analysis remained grounded in the data, with space for inductive insights and context-specific nuances to emerge organically. The framework served to enrich, rather than constrain, the thematic development (ST, ALR).

Finally, the fifth step involved writing up the themes in an integrated narrative format. Each theme was structured to reflect the lived realities of participants while highlighting the broader cognitive, social, and environmental dynamics that shape vaccination behavior. The final themes balanced both individual experiences and structural influences, offering a comprehensive picture of vaccine hesitancy and acceptance in wartime Ukraine. Thematic decisions were reviewed and cross-validated across research team members to ensure interpretive rigor and consistency (ST, ALR).

### Ethics considerations

The study received ethical approval from three institutions: the Bioethics Committee of Ivan Boberskyi Lviv State University of Physical Culture (protocol No. 24, dated December 20, 2024), Poltava Medical University in Ukraine (approval No. 227, dated May 23, 2024), and Rutgers University in the United States (protocol #Pro2024001126). All procedures adhered to the principles of the Declaration of Helsinki. Written informed consent was obtained from all participants before any study procedures were initiated, and participants were informed that they could withdraw from the study at any time. Data collection was carried out in accordance with all relevant ethical guidelines and regulatory requirements in both Ukraine and the United States.

In addition to the formalities of ethical clearance, the authors have considered additional issues. Doing research in conflict-afflicted settings requires deep grounding in local culture and institutions. Our author team includes Ukrainian health authorities and Ukrainian researchers based in Ukraine. Our participants’ vulnerability and their benefit are of our utmost concern, and the authors share the results amply to inform local decision-making and policy. This study did not present itself as an opportunity for any financial gain and ensured anonymity while still raising the participants’ voices.

## Findings

Participants represented a range of perspectives on childhood vaccination, including parents who generally supported vaccination, those who expressed uncertainty or hesitation, and a smaller number who reported delaying or questioning certain vaccines. While many participants described continued trust in pediatric recommendations, some highlighted concerns related to safety, access, or institutional uncertainty during the war.

### Social media makes me fear vaccines

Digital platforms played a significant role in shaping and amplifying vaccine fears, with many mothers describing how fear-driven narratives influenced them more because they are very visual and feeling emotional. Misinformation, particularly surrounding anecdotes of vaccine injuries or links to autism, spread rapidly through viral videos and posts. These stories, often emotionally resonant, left a stronger impression than official health messaging. As our participants mentioned:

*A single viral video can undo months of health education. Parents see one dramatic case online and suddenly question everything we’ve told them. It’s hard to fight fear with facts when emotions are involved.* (Physician 5)

*I’ve seen scary stories on Facebook about children harmed by vaccines. After watching those videos*,* even though I know some of them might not be true*,* I still can’t stop thinking about them. It really made me hesitate.* (Mother, 28)

Participants expressed that social media is a space where people can style themselves experts to create a polarizing environment in which constructive dialogue is rare. Online discussions about vaccines often devolved into arguments and opinion sharing, making it difficult to distinguish which sources were credible. This contributed to the confusion and mistrust parents felt while navigating vaccine decisions for the first time. Many mothers relied heavily on social media for health information, despite the expressed concerns about its quality. Platforms like Facebook, Instagram, and Telegram were the main sources of information for understanding vaccine requirements, risks, and trends. Reportedly, peers and other community members that share fear-based content influences decision making processes more heavily, especially for first-time parents. Social media also has potential, according to our participants. It can be a space for positive engagement. This was especially true for mothers with more education or with higher health literacy, who can more easily identify pro-vaccine groups and health campaigns. Although these are less visible, participants that accessed them described them as helpful.

*Unfortunately*,* accounts sharing misinformation often have far more followers than official pages with accurate information. For example*,* a well-known influencer might share unverified claims about vaccines*,* and because they have a large audience*,* their misinformation spreads quickly. Despite efforts to collaborate with such individuals*,* they often don’t want to cooperate.* (Physician 11)

*Usually*,* I don’t trust social media*,* but I read information on Facebook or YouTube from reputable doctors with experience. If they recommend vaccination*,* I trust them.* (Mother, 36)

### Survival is a priority during war and displacement

For many families, the ongoing war in Ukraine adds urgency to vaccine practices. Mothers reported making decisions in unstable and destabilizing environments where the immediate demands of survival, including securing shelter, food, and safety overtook routine healthcare. As a result, vaccination was often delayed or deprioritized in the face of relocation, displacement, or systemic breakdowns.

*I think the availability of vaccines has decreased during the war. Also storage of vaccines due to power outages caused by the war*,* made me think if this vaccine is really safe?! And for my family staying alive was the first priority so we missed kids’ vaccines.“* (Mother, 40)

This was not uncommon, and a physician shared

*Many children and families fled abroad or to western regions of Ukraine*,* disrupting the vaccination calendar… While some received vaccines abroad*,* others delayed them*,* waiting to return home… Overall*,* this led to a decrease in vaccination coverage initially*,* though rates are gradually recovering as people return and reengage with family doctors.* (Physician;17)

Our participants pointed out that access to vaccines was inconsistent because of the war and its effect on infrastructure and supply chains. In addition, clinics were downsizing, reducing capacity, or closing all together. Health staff were relocated or unavailable. Vaccine shortages became common, and both mothers and providers reported families could not follow through with vaccine schedules. When they were available, the uncertainty of travel alongside the possibility of air raids, especially in unsafe areas, affected their decision making. Health facilities were avoided by families because they are perceived as targeted for attack. This was true even for families that were willing to follow scheduled vaccines, because they felt confronted with unsurmountable barriers. The war also led to record-keeping challenges. Many families lost access to vaccination cards or were unsure of which doses their children had received. This created logistical confusion and made continuity of care difficult when moving between cities or into neighboring countries. For some, the lack of documentation resulted in starting the vaccine process over again.

Yet, despite the chaos, several mothers expressed strong commitment to vaccinating their children, even in unsafe or uncertain circumstances. Some traveled significant distances or endured long wait times to access health services.

*Even during the war*,* I didn’t stop vaccinating my children. I knew it was important. Sometimes we had to wait hours or travel far*,* but I kept going because I want to protect them. The trauma of war made me even more sure that I had to do everything I could to keep them safe.* (Mother; 31)

*At the beginning of the war*,* yes*,* there were vaccine shortages. It was hard to get appointments*,* and some clinics were closed or had no staff. But I still made sure all my child’s vaccinations were completed. We had to wait*,* and sometimes we didn’t know if the vaccines were available that day*,* but I kept going back. My only concern was about the quality of the vaccines during the war*,* whether they were stored properly or still safe. But I didn’t stop. I believe vaccination is still important*,* even in a war. People are still vaccinating their children just like before.* (Mother; 40)

### I am afraid of the effects of the vaccines

Many mothers expressed fear and concern over the negative consequences associated with vaccination. For some, there was a preference for natural immunity. Health was something best preserved through environmental control, clean homes, nutritious food, and exposure to natural elements, not biomedical interventions. Vaccines were perceived as artificial, unnecessary intrusions, especially for seemingly healthy children.

*I believe if children eat healthy*,* stay active*,* and we keep the house clean*,* they won’t get sick. That’s how we were raised*,* and we didn’t need so many shots. I worry about what’s in the vaccines*,* chemicals*,* preservatives*,* and how they affect small bodies. Sometimes I feel it’s safer to rely on the body’s natural strength. Why give a vaccine if the child is already healthy and strong? I’m not against medicine*,* but I don’t think it should be the first solution.* (Mother; 31)

Traumatic memories of being vaccinated, including experiencing side effects like fainting or general malaise, as well as being forced into the vaccine, were part of what mothers reflected on when making decisions for their children.

*When I was a child*,* I fainted after getting a vaccine at school. I remember feeling sick for days*,* and no one really explained what had happened. They just gave us the shots without asking or telling us why. That experience has stayed with me. Now*,* as a mother*,* I’m very careful. I don’t want my child to go through what I did. I ask a lot of questions because I don’t want to repeat the same mistakes.* (Mother; 36)

Mothers frequently mentioned anxiety about side effects and a lack of transparency regarding vaccine ingredients, including preservatives and additives. Experiences of younger children becoming sick after a vaccine, including running a fever, but also fears of longer-term mobility or neurological issues. Many questioned if the health authorities were able to secure proper storage and follow all handling protocols. This was especially true now, when health infrastructure is under strain and confidence in the capacity of the health authorities is low.

The perceived lack of proper communication from healthcare providers further exacerbated vaccine fears. Mothers often felt rushed during appointments, receiving little or no explanation about what the vaccine was for or its potential side effects. The absence of thorough pediatric assessments prior to vaccination, combined with limited dialogue, led many to feel alienated rather than reassured.

*I feel pressured to vaccinate my child without proper explanations. Doctors are often in a hurry*,* and I don’t get the chance to ask questions. I want to understand what’s going into my child’s body*,* but there’s no time*,* no discussion.* (Mother; 31)

*Doctors don’t explain children’s vaccines clearly because they’re too rushed. Mothers are left confused*,* and that’s where doubts begin. We need to slow down and talk to parents properly.* (Physician 7)

### Building trust in vaccines through professionals and institutions

In a context where participants felt surrounded by fear and misinformation, many emphasized the importance of trusted healthcare providers and how they have helped in shaping their vaccine decisions. Personal relationships with pediatricians played was important for relieving concerns from mothers. When physicians took the time to explain, listen, and recommend vaccines, mothers felt more inclined to follow their guidance. Even in the presence of online misinformation or community-level skepticism, a trusted pediatrician often served as the final authority in decision-making.

*I rely more on my pediatrician than social media for vaccine advice. When I have doubts*,* I talk to our doctor*,* and she explains everything. That helps me feel more confident. Even if I see scary things online*,* I still go with what she says because she knows my child”.* (Mother, 29)

Participants also recognized the protective value of vaccines, particularly in shielding children from serious diseases. Vaccination was widely acknowledged as a preventive tool, especially among those with prior experience of illness or awareness of local outbreaks. Mothers expressed a strong desire to protect their children from suffering, and despite underlying fears, this protective instinct motivated many to continue with immunization schedules.

*Some parents come in with notes and want every vaccine available. They’ve read about the risks but still choose to vaccinate their kids. I think they trust the protection more than they fear the side effects.* (Physician, 4)

*I vaccinate because I want to protect my child from dangerous diseases. I’ve seen what can happen when children aren’t vaccinated. Even though I worry about side effects*,* I’d rather take that small risk than see my child suffer from something serious.* (Mother, 30)

Participants expressed support for educational and community-based strategies aimed at increasing vaccine confidence. Clear and transparent information was highly valued. Parents wanted to know what vaccines contained, why they were necessary, and what risks were involved. When these explanations were omitted, doubt and resistance grew. Inclusive messaging that balanced risks with benefits, acknowledged historical concerns, and used everyday language was seen as the most credible. Storybooks, cartoons, health fairs, and other outreach tools were seen as effective ways to demystify vaccines and engage parents in non-threatening, accessible formats. A physician mentioned:

*Children’s storybooks about vaccines make a difference. We also use cartoons and visual materials to help explain things to adolescents. These tools really help parents understand without feeling attacked or judged.* (Mother, 30)

## Discussion

This study explored the ways mothers (46) and healthcare professionals (22) living and working in Ukraine perceive and respond to information fragmentation on childhood vaccination and health system limitations under the stressors of war. Four themes emerged: (1) social media makes me fear vaccines; (2) Survival is a priority first during war and displacement. (3) I am afraid of the effects of the vaccines, and (4) building trust in vaccines through professionals and institutions. We look at the ways mothers and healthcare professionals in Ukraine navigate childhood vaccination decisions under the extreme and overlapping pressures of war, displacement, and information disorder. Our findings reveal a deeply entangled network of factors, emotional, relational, institutional, and structural, that contribute to vaccine hesitancy and resilience alike. These challenge one-dimensional models of hesitancy by underscoring how trust, trauma, uncertainty, and digital influence operate within a collapsing health infrastructure.

The central role of social media in shaping vaccine-related perceptions among our participants was a strong theme in our findings. Many mothers described how their confidence in vaccination was diminished by emotionally charged stories encountered online, especially videos that depicted children harmed by vaccines. These narratives often overpowered formal health messaging, which was seen as abstract, delayed, or emotionally disconnected. The centrality of social media in shaping vaccine-related perceptions among our participants reflects how interpersonal networks and digital environments fill the vacuum left by disrupted formal communication systems. According to the Multilevel Framework of Vaccine Behavior, such interpersonal influences, including peer narratives and viral content, play a heightened role when institutional channels are weakened, as is often the case in conflict-affected settings. In our findings, emotionally charged stories encountered online, especially videos showing children allegedly harmed by vaccines, overpowered official health messaging. These informal digital sources became proxies for trusted information, particularly when formal communication was perceived as abstract, delayed, or emotionally disconnected. This finding aligns with scholarship showing how fear-based misinformation spreads faster than facts, particularly when delivered in compelling visual formats [54, 55].

The nature of social media, algorithmically tailored and participatory, creates echo chambers that reinforce pre-existing beliefs [55]. This dynamic amplifies risk perceptions for less educated or digitally unprepared users [52]. As noted by Cinelli et al., [10], social platforms often reward content that evokes outrage or fear, making misinformation both virulent and persistent [10]. In our study, mothers with higher health literacy or access to verified medical professionals were better able to interpret online content critically. However, younger or first-time mothers without these resources described themselves as overwhelmed and paralyzed. The relationship between health literacy, stress, and digital exposure thus becomes central in understanding how vaccine beliefs evolve under duress [29, 39]. The institutional silence that often accompanied this digital chaos created a vacuum of information that may have allowed more actors to disseminate false or unverified information successfully. As participants explained, few official channels countered misinformation with consistency or clarity. This absence of visible public health communication enabled false narratives to fill the vacuum, as echoed in studies on pandemic response and vaccine uptake [27]. Loomba et al., [27]. A study by Loomba et al., [27] conducted in the United Kingdom (UK) and the United States of America (USA) showed that exposure to COVID-19 vaccine misinformation reduced the intent to vaccinate by over 6% points in both countries. Moreover, in the US, partisan polarization and erosion of institutional trust exacerbated the reach and impact of misinformation [20]. This comparative perspective underscores that institutional silence or inconsistency, whether caused by war or political fragmentation, functions as a critical enabler of misinformation. In Ukraine, these challenges are further amplified by trauma, displacement, and the collapse of health infrastructure, creating a uniquely high-risk setting where even motivated caregivers struggle to find credible and reassuring sources.

Vaccine hesitancy in the communities that were part of our sample was not a product of ignorance, but of lived trauma. Participants’ narratives of past vaccination injuries, childhood fainting episodes, and forced medical procedures resurfaced as powerful emotional residues influencing current decisions. These findings resonate with research linking adverse childhood healthcare experiences with later mistrust in medical systems [30]. The emotional residue of trauma intensified under the pressure of caregiving in war, where medical decisions carry existential weight [37]. Mothers did not distrust vaccines in isolation; they distrusted a crumbling system. The erosion of infrastructure, disruptions to cold chains, and staff shortages contributed to doubts about vaccine safety and handling. Many participants explicitly questioned whether wartime vaccines were adequately stored, expired, or administered correctly. These perceptions mirrored findings from post-Ebola West Africa, where systemic breakdowns damaged vaccine trust long after the outbreak [53]. Importantly, this trust gap was not universal. Many mothers continued to trust their personal pediatricians, even while losing faith in the broader system. This highlights a relational–institutional trust divide that has been documented in other fragile settings [5, 41]. However, this relational trust was also fragile, susceptible to relocation, provider turnover, or inconsistent care. When mothers lost access to a known doctor, restarting the vaccine decision-making process felt emotionally and logistically overwhelming. This underscores how personalized, relational trust can serve as a temporary buffer against systemic failure, but it is not a substitute for sustained institutional reliability.

Communication emerged as a central buffer against fear, but only when it was meaningful and bi-directional. Rushed consultations, limited time for questions, and one-size-fits-all advice left parents feeling alienated from the health system and from providers. This phenomenon is widely reported in vaccine literature, with studies emphasizing that perceived provider empathy and clear explanations are crucial in building confidence [12, 24]. Participants who felt heard, respected, and informed by providers were more likely to follow through with vaccination, even when doubts persisted. Similar findings have been reported in Nigeria and the Philippines, where provider communication style, especially listening and emotional validation, directly influenced parental willingness to vaccinate [6, 8].

The war in Ukraine created a landscape of chronic instability that directly undermined vaccine access and confidence. Participants described skipping or delaying vaccines due to safety risks, logistical breakdowns, and fear of attending clinics that might be targeted. These disruptions are consistent with global evidence that armed conflict reduces vaccination coverage by damaging infrastructure, displacing communities, and straining cold chains [15]. Similar patterns have been observed in Syria, where the collapse of health infrastructure led to sharp declines in measles and polio immunization [50], and in Venezuela, where political and economic turmoil disrupted cold chains and led to the resurgence of vaccine-preventable diseases like diphtheria and measles [42]. In South Sudan, recurrent conflict and displacement were linked to significant drops in immunization coverage, with humanitarian access constraints compounding the problem [16]. These cases show that in conflict-affected settings, even when demand exists, structural breakdowns and perceived danger create formidable barriers to vaccination. In such environments, parents often make difficult trade-offs. Choosing not to vaccinate was frequently a form of rational prioritization rather than ideological opposition. Families focused first on securing food, shelter, and physical safety. This reframing is important: describing these mothers as “non-compliant” ignores the moral logic of their decisions. Scholars have advocated for crisis-responsive public health models that interpret delays not as resistance but as adaptive responses to trauma [12].

Even when mothers wished to vaccinate, displacement created barriers. Many lost vaccination records or moved across borders, leading to confusion about dose schedules. Some had to restart vaccinations from scratch, while others lacked trust in host-country systems. These record-keeping challenges mirror findings from refugee studies in Syria, Afghanistan, and South Sudan [2, 14, 31, 36].

A recurring insight was that some mothers viewed vaccines as curative rather than preventive. This perception often stemmed from witnessing the visible consequences of illness or experiencing post-vaccination health changes. When vaccines did not produce immediate or visible benefits, some questioned their efficacy. This misalignment between public health logic and lay expectations reflects a common challenge in preventive medicine [34]. Public health communication often assumes that people understand vaccines as anticipatory tools. Yet in contexts where disease absence is taken for granted, and risk is abstract, this assumption falters. Effective messaging may need to make the preventive logic of vaccines more concrete, linking immunization to stories of protection, survival, and resilience. Visual materials, health fairs, and community-based storytelling, described positively by participants, are promising tools in this regard. Studies in Sierra Leone and Lebanon suggest these approaches are especially effective when they center lived experience and involve trusted community actors [19, 49, 51].

At the heart of these decisions lies maternal agency, the capacity to act, interpret, and protect within constrained environments [21, 33]. Despite overwhelming barriers, many mothers persevered in vaccinating their children, sometimes walking miles, waiting hours, or persisting through shortages. This underscores the importance of designing health interventions that support, rather than circumvent, maternal decision-making. The literature supports approaches that empower caregivers through education, peer networks, and access to clear, empathetic information [27, 40] Still, agency is not evenly distributed. Mothers with lower education, higher stress, or fewer social supports were more vulnerable to fear-driven narratives and less able to critically assess conflicting information. This inequity reinforces calls for equity-informed vaccine promotion, which tailors strategies to account for digital, psychological, and structural disadvantage [23, 32]. Equally, the state bears responsibility. Trust cannot be restored solely through interpersonal relationships. Institutional accountability, timely messaging, visible investment, and respectful engagement are essential. In the absence of such efforts, vaccine confidence may remain vulnerable to the next disruption, war, or wave of misinformation.

The findings also highlight an important epistemic tension between institutional health messaging and parents’ everyday experiences of risk decision-making. Public health institutions typically communicate vaccination through standardized scientific evidence and population level risk assessments. However, parents often interpret vaccination decisions through lived experiences, personal networks, and contextual uncertainties. As Eyal (2019) argues, contemporary health controversies frequently involve a “crisis of expertise,” where individuals navigate competing sources of knowledge and attempt to reconcile institutional authority with personal judgment. Similarly, shows that some parents frame vaccine decision-making as a form of responsible and attentive parenting, grounded in individualized risk evaluation rather than passive acceptance of institutional guidance. Our findings reflect similar dynamics. mothers often did not reject vaccines outright but described navigating uncertainty by weighing institutional recommendations against everyday experiences, social relationships, and wartime disruptions. In this context, vaccine hesitancy can be understood less as a simple rejection of science and more as an attempt to manage risk in a complex and unstable environment.

## Conclusion

This study set out to answer that question in the context of wartime Ukraine, where the overlapping pressures of displacement, trauma, digital misinformation, and institutional disruption converge on the most intimate site of health decision-making: the care of children. Our findings show that vaccine hesitancy in such settings is not simply a matter of lacking information or harboring irrational fears. Instead, it emerges as a deeply contextual, emotionally driven response to lived experiences of insecurity, abandonment, and fractured trust. Mothers weigh not only the clinical risks and benefits of vaccines, but also their symbolic and moral meanings, what they represent in a time of instability. In this environment, vaccine decisions are shaped as much by survival priorities, memory, and social influence as by provider recommendations. Fear circulates rapidly through digital channels, while trust is built slowly, often hinging on personal interactions with healthcare workers who take time to listen and explain. Hesitancy, in this context, can be seen not as a fixed position but as a fluid state, negotiated and re-negotiated through relationships, experiences, and institutional encounters.

Addressing vaccine hesitancy in conflict-affected settings like Ukraine requires more than supply chains and schedules. It demands communication strategies that recognize emotional reasoning, interventions that rebuild institutional trust, and policies that support, not override, maternal agency. Only by grounding public health efforts in the lived realities of those making these decisions can we foster vaccine confidence amidst a crisis.

## Data Availability

Original datasets will be available from the corresponding author upon request.

## References

[1] Arghittu A, Deiana G, Dettori M, Castiglia P. Vaccination, public health and health communication: a network of connections to tackle global challenges. Vaccines. 2025;13(3). Multidisciplinary Digital Publishing Institute (MDPI). 10.3390/vaccines13030245. 10.3390/vaccines13030245 PMC11945708 40266117

[2] Audi MN, Mwenda KM, Wei G, Lurie MN. Healthcare accessibility in preconflict Syria: a comparative spatial analysis. BMJ Open. 2022;12(5). 10.1136/bmjopen-2021-059210. 10.1136/bmjopen-2021-059210 PMC9073410 35508340

[3] Badovinac SD, Flora DB, Edgell H, Flanders D, Garfield H, Weinberg E, Savlov D, Riddell P, R. R. Caregivers’ physiological responses during toddler vaccinations: associations with psychological and behavioral responses. J Pediatr Psychol. 2025;50(4):307–16. 10.1093/jpepsy/jsae095. 39579363 10.1093/jpepsy/jsae095 PMC12013813

[4] Bedford H, Attwell K, Danchin M, Marshall H, Corben P, Leask J. Vaccine hesitancy, refusal and access barriers: the need for clarity in terminology. Vaccine. 2018;36(44):6556–8. 10.1016/j.vaccine.2017.08.004. 28830694 10.1016/j.vaccine.2017.08.004

[5] Benin AL, Wisler-Scher DJ, Colson E, Shapiro ED, Holmboe ES. Qualitative analysis of mothers’ decision-making about vaccines for infants: the importance of trust. Pediatrics. 2006;117(5):1532–41. 10.1542/peds.2005-1728. 16651306 10.1542/peds.2005-1728

[6] Bosch-Capblanch X, Banerjee K, Burton A. Unvaccinated children in years of increasing coverage: how many and who are they? Evidence from 96 low- and middle-income countries. Trop Med Int Health. 2012;17(6):697–710. 10.1111/j.1365-3156.2012.02989.x. 22943300 10.1111/j.1365-3156.2012.02989.x

[7] Budigan Ni H, de Broucker G, Patenaude BN, Dudley MZ, Hampton LM, Salmon DA. Economic impact of vaccine safety incident in Ukraine: the economic case for safety system investment. Vaccine. 2023;41(1):219–25. 10.1016/j.vaccine.2022.11.004. 36435704 10.1016/j.vaccine.2022.11.004

[8] Butler R, MacDonald NE, Eskola J, Liang X, Chaudhuri M, Dube E, Gellin B, Goldstein S, Larson H, Manzo ML, Reingold A, Tshering K, Zhou Y, Duclos P, Guirguis S, Hickler B, Schuster M. Diagnosing the determinants of vaccine hesitancy in specific subgroups: the guide to Tailoring Immunization Programmes (TIP). Vaccine. 2015;33(34):4176–9. 10.1016/j.vaccine.2015.04.038. 25896376 10.1016/j.vaccine.2015.04.038

[9] Ciccacci F, Ruggieri E, Scarcella P, Moramarco S, Carestia M, Di Giovanni D, Silaghi LA, Altan D, A. M., Orlando S. Between war and pestilence: the impact of armed conflicts on vaccination efforts: a review of literature. Front Public Health. 2025;13. Frontiers Media SA. 10.3389/fpubh.2025.1604288. 10.3389/fpubh.2025.1604288 PMC12259657 40666147

[10] Cinelli M, Quattrociocchi W, Galeazzi A, Valensise CM, Brugnoli E, Schmidt AL, Zola P, Zollo F, Scala A. The COVID-19 social media infodemic. Sci Rep. 2020;10(1). 10.1038/s41598-020-73510-5. 10.1038/s41598-020-73510-5 PMC7538912 33024152

[11] Costantino V, MacIntyre CR. Impact of vaccine coverage and disruption to health services on COVID-19 in Ukraine. Sci Rep. 2024;14(1). 10.1038/s41598-024-57447-7. 10.1038/s41598-024-57447-7 PMC11208616 38926448

[12] Dubé E, Laberge C, Guay M, Bramadat P, Roy R, Bettinger J. Vaccine hesitancy: an overview. Hum Vaccin Immunother. 2013;9(8):1763–1773. 10.4161/hv.24657. 10.4161/hv.24657 PMC3906279 23584253

[13] Dvergsdal ET, Campbell S, Lerstad ND, Greve-Isdahl M, Labberton AS, Hansen BT, Meijerink H. Low childhood vaccination coverage among Ukrainian Refugees in Norway. A nationwide, register-based cohort study, 2022–2023. J Immigr Minor Health. 2025. 10.1007/s10903-025-01725-7. 40668470 10.1007/s10903-025-01725-7 PMC12669341

[14] Fozouni L, Weber C, Lindner AK, Rutherford GW. Immunization coverage among refugee children in Berlin. J Global Health. 2019;9(1). 10.7189/JOGH.09.010432. 10.7189/jogh.09.010432 PMC6571106 31217960

[15] Gallagher KE, LaMontagne DS, Watson-Jones D. Status of HPV vaccine introduction and barriers to country uptake. Vaccine. 2018;36(32):4761–7. 10.1016/j.vaccine.2018.02.003. 29580641 10.1016/j.vaccine.2018.02.003

[16] Grundy J, Biggs BA. The impact of conflict on immunisation coverage in 16 countries. Int J Health Policy Manage. 2019;8(4):211–21. 10.15171/IJHPM.2018.127. 10.15171/ijhpm.2018.127 PMC6499911 31050966

[17] Hill M, Vanderslott S, Volokha A, Pollard AJ. Addressing vaccine inequities among Ukrainian refugees. Lancet Infect Dis. 2022;22:935–6. Elsevier Ltd. 10.1016/S1473-3099(22)00366-8. 10.1016/S1473-3099(22)00366-8 PMC9221088 35752178

[18] Holford D, Anderson EC, Biswas A, Garrison A, Fisher H, Brosset E, Gould VC, Verger P, Lewandowsky S. Healthcare professionals’ perceptions of challenges in vaccine communication and training needs: a qualitative study. BMC Prim Care. 2024;25(1). 10.1186/s12875-024-02509-y. 10.1186/s12875-024-02509-y PMC11265004 39033114

[19] Jalloh MF, Sengeh P, Ibrahim N, Kulkarni S, Sesay T, Eboh V, Jalloh MB, Pratt SA, Webber N, Thomas H, Kaiser R, Singh T, Prybylski D, Omer SB, Brewer NT, Wallace AS. Association of community engagement with vaccination confidence and uptake: a cross-sectional survey in Sierra Leone, 2019. J Glob Health. 2022;12. 10.7189/jogh.12.04006. 10.7189/jogh.12.04006 PMC8876869 35265325

[20] Jamieson KH, Albarracin D. The relation between media consumption and misinformation at the outset of the SARS-CoV-2 pandemic in the US. Harv Kennedy School Misinformation Rev. 2020;1(3). 10.37016/mr-2020-012. 10.37016/mr-2020-012 PMC12424044 40948887

[21] Kbaier D, Kane A, McJury M, Kenny I. Prevalence of health misinformation on social media—challenges and mitigation before, during, and beyond the COVID-19 pandemic: scoping literature review. J Med Internet Res. 2024;26:e38786. 10.2196/38786. 39159456 10.2196/38786 PMC11369541

[22] Larson HJ, Gakidou E, Murray CJL. The vaccine-hesitant moment. N Engl J Med. 2022;387(1):58–65. 10.1056/nejmra2106441. 35767527 10.1056/NEJMra2106441 PMC9258752

[23] Larson HJ, Jarrett C, Eckersberger E, Smith DMD, Paterson P. Understanding vaccine hesitancy around vaccines and vaccination from a global perspective: a systematic review of published literature, 2007–2012. Vaccine. 2014;32:2150–9. Elsevier BV. 10.1016/j.vaccine.2014.01.081. 10.1016/j.vaccine.2014.01.081 24598724

[24] Leask J, Kinnersley P, Jackson C, Cheater F, Bedford H, Rowles G. Communicating with parents about vaccination: a framework for health professionals. BMC Pediatr. 2012;12. BioMed Central Ltd. 10.1186/1471-2431-12-154. 10.1186/1471-2431-12-154 PMC3480952 22998654

[25] Lecce M, Milani GP, Agostoni C, D’Auria E, Banderali G, Biganzoli G, Castellazzi L, Paramithiotti C, Salvatici E, Tommasi P, Zuccotti GV, Marchisio P, Castaldi S. Caregivers’ intention to vaccinate their children under 12 years of age against COVID-19: a cross-sectional multi-center study in Milan, Italy. Front Pead. 2022;10. 10.3389/fped.2022.834363. 10.3389/fped.2022.834363 PMC9196897 35712618

[26] Lewtak K, Mazur J, Dwyer H, Sochoń-Latuszek A, Atif A, Maciejewski T, Kleszczewska D. Determinants of Ukrainian mothers’ intentions to vaccinate their children in Poland: a cross-sectional study. Vaccines. 2025;13(3). 10.3390/vaccines13030325. 10.3390/vaccines13030325 PMC11945471 40266242

[27] Loomba S, de Figueiredo A, Piatek SJ, de Graaf K, Larson HJ. Measuring the impact of COVID-19 vaccine misinformation on vaccination intent in the UK and USA. Nat Hum Behav. 2021;5(3):337–48. 10.1038/s41562-021-01056-1. 33547453 10.1038/s41562-021-01056-1

[28] MacDonald NE, Eskola J, Liang X, Chaudhuri M, Dube E, Gellin B, Goldstein S, Larson H, Manzo ML, Reingold A, Tshering K, Zhou Y, Duclos P, Guirguis S, Hickler B, Schuster M. Vaccine hesitancy: definition, scope and determinants. Vaccine. 2015;33(34):4161–4. 10.1016/j.vaccine.2015.04.036. 25896383 10.1016/j.vaccine.2015.04.036

[29] Mackert M, Mabry-Flynn A, Champlin S, Donovan EE, Pounders K. Health literacy and health information technology adoption: the potential for a new digital divide. J Med Internet Res. 2016;18(10). 10.2196/jmir.6349. 10.2196/jmir.6349 PMC5069402 27702738

[30] Madigan S, Wade M, Plamondon A, Maguire JL, Jenkins JM. Maternal adverse childhood experience and infant health: biomedical and psychosocial risks as intermediary mechanisms; 2017. 10.1016/j.jpeds. 10.1016/j.jpeds.2017.04.052 28549634

[31] Mahimbo A, Seale H, Smith M, Heywood A. Challenges in immunisation service delivery for refugees in Australia: a health system perspective. Vaccine. 2017;35(38):5148–55. 10.1016/j.vaccine.2017.08.002. 28802753 10.1016/j.vaccine.2017.08.002

[32] McCaffery KJ, Morony S, Muscat DM, Smith SK, Shepherd HL, Dhillon HM, Hayen A, Luxford K, Meshreky W, Comings J, Nutbeam D. Evaluation of an Australian health literacy training program for socially disadvantaged adults attending basic education classes: study protocol for a cluster randomised controlled trial. BMC Public Health. 2016;16(1). 10.1186/s12889-016-3034-9. 10.1186/s12889-016-3034-9 PMC4884424 27233237

[33] Mezen MK, Lemlem GA, Biru YB, Yimer AM. Association of war with vaccination dropout among children younger than 2 years in the North Wollo Zone, Ethiopia. JAMA Netw Open. 2023;6(2):e2255098–2255098. 10.1001/jamanetworkopen.2022.55098. 36749587 10.1001/jamanetworkopen.2022.55098 PMC10408260

[34] Miles Braun M, Patriarca PA, Ellenberg SS. Syncope after immunization; 1997. 10.1001/archpedi.1997.02170400041007 9080932

[35] Mobilization WH, Organization S, Team T. Dengue and DHF (p. WHO/CDS/CPE/SMT/2001.9). World Health Organization.

[36] Mohammed RN, Khawari A, Shaguy JA, Abouzied A. A GIS-based approach to identifying communities underserved by primary health care services—An Afghanistan case study. Front Public Health. 2023;11. 10.3389/fpubh.2023.1209986. 10.3389/fpubh.2023.1209986 PMC10552865 37809002

[37] Moreira da Cunha N, Tzirita S, Gobbo E, van Herzig S. Factors influencing adolescents’ decision-making about COVID-19 vaccination: a systematic review with qualitative synthesis. Front Public Health. 2025;13. Frontiers Media SA. 10.3389/fpubh.2025.1563677. 10.3389/fpubh.2025.1563677 PMC12116342 40438050

[38] Nnadi C, Etsano A, Uba B, Ohuabunwo C, Melton M, Wa Nganda G, Esapa L, Bolu O, Mahoney F, Vertefeuille J, Wiesen E, Durry E. Approaches to vaccination among populations in areas of conflict. J Infect Dis. 2017;216:S368–72. 10.1093/infdis/jix175. 28838202 10.1093/infdis/jix175 PMC5754212

[39] Nutbeam D, E Lloyd J. Understanding and responding to health literacy as a social determinant of health. Annual Rev Public Health. 2025;42:2020. 10.1146/annurev-publhealth. 10.1146/annurev-publhealth-090419-102529 33035427

[40] Odone A, Tillmann T, Sandgren A, Williams G, Rechel B, Ingleby D, Noori T, Mladovsky P, McKee M. Tuberculosis among migrant populations in the European Union and the European Economic Area. Eur J Pub Health. 2015;25(3):506–12. 10.1093/eurpub/cku208. 25500265 10.1093/eurpub/cku208 PMC4440450

[41] Ozawa S, Stack ML. Public trust and vaccine acceptance-international perspectives. Hum Vaccin Immunother. 2013;9(8):1774–1778. 10.4161/hv.24961. 10.4161/hv.24961 PMC3906280 23733039

[42] Paniz-Mondolfi AE, Tami A, Grillet ME, Márquez M, Hernández-Villena J, Escalona-Rodríguez MA, Blohm GM, Mejías I, Urbina-Medina H, Rísquez A, Castro J, Carvajal A, Walter C, López MG, Schwabl P, Hernández-Castro L, Miles MA, Hotez PJ, Lednicky J, Oletta J. Resurgence of vaccine-preventable diseases in Venezuela as a regional public health threat in the Americas. Emerg Infect Dis. 2019;25(4):625–32. 10.3201/eid2504.181305. 30698523 10.3201/eid2504.181305 PMC6433037

[43] Paquin V, Miconi D, Aversa S, Johnson-Lafleur J, Côté S, Geoffroy M-C, Gülöksüz S. Social and mental health pathways to institutional trust: a cohort study. Soc Sci Med. 2025;379:118199. 10.1016/j.socscimed.2025.118199. 40382867 10.1016/j.socscimed.2025.118199

[44] Parrish-Sprowl J, Thomson A, Johnson RD, Parrish-Sprowl S. The AIMS approach: regulating receptivity in patient-provider vaccine conversations. Front Public Health. 2023;11. 10.3389/fpubh.2023.1120326. 10.3389/fpubh.2023.1120326 PMC10273204 37333542

[45] Petakh P, Tymchyk V, Kamyshnyi O. Communicable diseases in Ukraine during the period of 2018–2023: Impact of the COVID-19 pandemic and war. Travel Med Infect Dis. 2024;60. 10.1016/j.tmaid.2024.102733. 10.1016/j.tmaid.2024.102733 38942160

[46] Rand. Invisible wounds of war: psychological and cognitive injuries, their consequences, and services to assist recovery. RAND Corporation. 2008. 10.7249/MG720.

[47] Ruiz-Incertis R, Tuñón-Navarro J. European institutional discourse concerning the Russian invasion of Ukraine on the Social Network X. Journalism Media. 2024;5(4):1646–83. 10.3390/journalmedia5040102.

[48] Sapienza A, Falcone R. The role of trust in COVID-19 vaccine acceptance: considerations from a systematic review. Int J Environ Res Public Health. 2023;20(1). MDPI. 10.3390/ijerph20010665. 10.3390/ijerph20010665 PMC9819668 36612982

[49] Shaarani I, Khadem S, Obeid M, Saadieh B, Serhal A, Zakkour K, Mohammad S, Berjaoui H, Izmirli N. Beliefs and attitudes of Syrian refugee mothers in Lebanon regarding children vaccination: a cross-sectional study. BMC Public Health. 2025;25(1). 10.1186/s12889-025-21290-w. 10.1186/s12889-025-21290-w PMC11715023 39780134

[50] Sharara SL, Kanj SS. War and infectious diseases: challenges of the Syrian Civil War; 2014. 10.1371/journal.ppat. 10.1371/journal.ppat.1004438 PMC4231133 25393545

[51] Sommers T, Dockery M, Burke N, D’Souza S, Troupe B, Agbonyinma T, Raghuram H, Hopkins KL, Kohlway E, Stojicic P, Bhan A. Building trust and equity in vaccine communication through community engagement. Hum Vacc Immunother. 2025l;21(1). Taylor and Francis Ltd. 10.1080/21645515.2025.2518636. 10.1080/21645515.2025.2518636 PMC12184151 40526370

[52] Songchon C, Wright G, Beevers L. Quality assessment of crowdsourced social media data for urban flood management. Comput Environ Urban Syst. 2021;90. 10.1016/j.compenvurbsys.2021.101690.

[53] Suk JE, Jimenez AP, Kourouma M, Derrough T, Baldé M, Honomou P, Kolie N, Mamadi O, Tamba K, Lamah K, Loua A, Mollet T, Lamah M, Nana Camara A, Prikazsky V. Post-ebola measles outbreak in lola, Guinea, January-June 2015. Emerg Infect Dis. 2016;22(6):1106–8. 10.3201/eid2206.151652. 27191621 10.3201/eid2206.151652 PMC4880080

[54] Vosoughi S, Roy D, Aral S. The spread of true and false news online. 2025. https://www.science.org. 10.1126/science.aap9559 29590045

[55] Wilson SL, Wiysonge C. Social media and vaccine hesitancy. BMJ Global Health. 2020;5(10). 10.1136/bmjgh-2020-004206. 10.1136/bmjgh-2020-004206 PMC7590343 33097547

[56] Wiysonge CS, Ndwandwe D, Ryan J, Jaca A, Batouré O, Anya BPM, Cooper S. Vaccine hesitancy in the era of COVID-19: could lessons from the past help in divining the future? Hum Vaccines Immunother. 2022;18(1):1–3. 10.1080/21645515.2021.1893062. 10.1080/21645515.2021.1893062 PMC8920215 33684019

